# Supplementary material for: DNA damage drives antigen diversification through mosaic Variant Surface Glycoprotein (VSG) formation in Trypanosoma brucei
Source: bioRxiv. 2024 Aug 30:2024.03.22.582209. Preprint. [Version 2] doi: 10.1101/2024.03.22.582209 (PMC11383311; doi:10.1101/2024.03.22.582209)
Supplement: 1 [file NIHPP2024.03.22.582209V2-supplement-1.pdf]

bioRxiv preprint doi: https://doi.org/10.1101/2024.03.22.582209; this version posted August 30, 2024. The copyright holder for this preprint (which was not certified by peer review) is the author/funder, who has granted bioRxiv a license to display the preprint in perpetuity. It is made available under aCC-BY 4.0 International license.

Supplemental figures

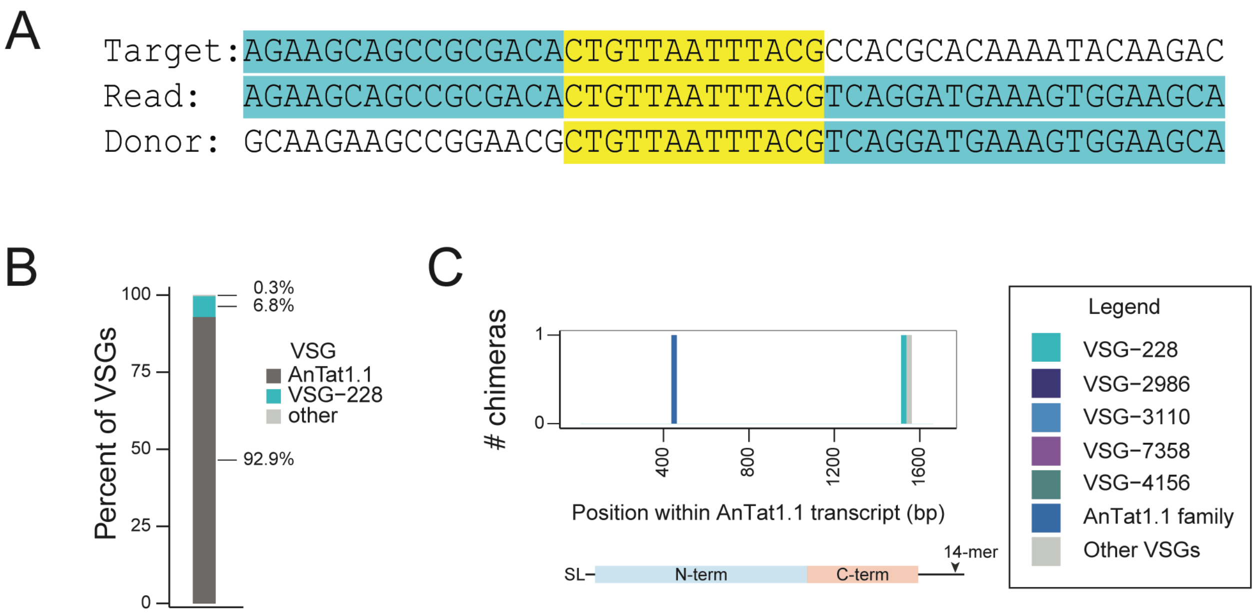

Supplemental Figure 1. Mosaic Recombination Site & VSG-AMP-seq Validation
A) A schematic of a recombination site. The region in yellow is the putative recombination site, shared by all 3 sequences. B) A stacked bar graph quantifying the mix of Lister427 parasites engineered to express either AnTat1.1 or VSG-228 as determined by VSG-seq. These VSGs are naturally absent from Lister427 parasites and therefore chimeric reads containing sequence from both VSGs must have arisen from error. C) A histogram showing the midpoint of chimeric recombination events misidentified as a mosaic VSGs by VSG-AMP-seq from the mixed parasite sample in Supplemental Figure 1B.

bioRxiv preprint doi: https://doi.org/10.1101/2024.03.22.582209; this version posted August 30, 2024. The copyright holder for this preprint (which was not certified by peer review) is the author/funder, who has granted bioRxiv a license to display the preprint in perpetuity. It is made available under aCC-BY 4.0 International license.

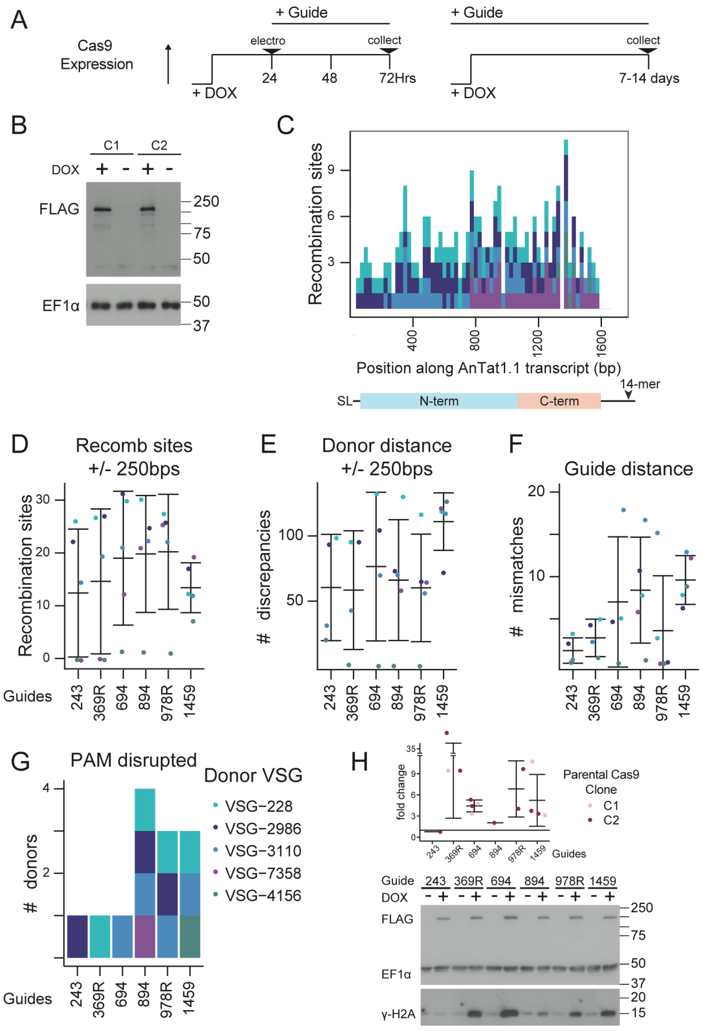

Supplemental Figure 2. Cas9 system and sgRNA design and analysis

A) A schematic of the Cas9 induction experiment. Transient electroporation shown on the left while constitutive guide expression to isolate mosaic clones shown at right. B) An immunoblot showing FLAG-tagged Cas9 induction 24 hours following doxycycline treatment (DOX). DMSO was used as a vehicle control. EF1α was used as a loading control. C) A histogram showing the midpoint of all possible recombination sites 5bps or longer between AnTat1.1 and its family members. D) Quantification of the number of recombination sites within 250bps up or downstream of the cut site for each donor VSG. Mean ± s.d. E) Quantification of the Levenshtein distance between AnTat1.1 and family members. This includes mismatches, insertions and deletions. Mean ± s.d. F) Quantification of the number of mismatches at the guide binding site between AnTat1.1 and family members. Mean ± s.d. G) Histogram of which donor VSGs can disrupt the PAM when used to repair AnTat1.1. H) Quantification of the induction of DNA damage following guide induction as measured by a proxy of DNA damage, γ-H2A phosphorylation, following 24 hours of induction. γ-H2A induction was normalized to EF1α loading and fold change of staining intensity was determined between doxycycline-induced and uninduced samples. DMSO was used as a vehicle control. Mean ± s.d. Below, a representative immunoblot of FLAG, EF1α, and γ-H2A from doxycycline induced clones and uninduced controls is shown. Multiple independently-generated clones were induced. SL = 5′ splice leader sequence, 14-mer = 3′ sequence conserved in all VSG transcripts

bioRxiv preprint doi: https://doi.org/10.1101/2024.03.22.582209; this version posted August 30, 2024. The copyright holder for this preprint (which was not certified by peer review) is the author/funder, who has granted bioRxiv a license to display the preprint in perpetuity. It is made available under aCC-BY 4.0 International license.

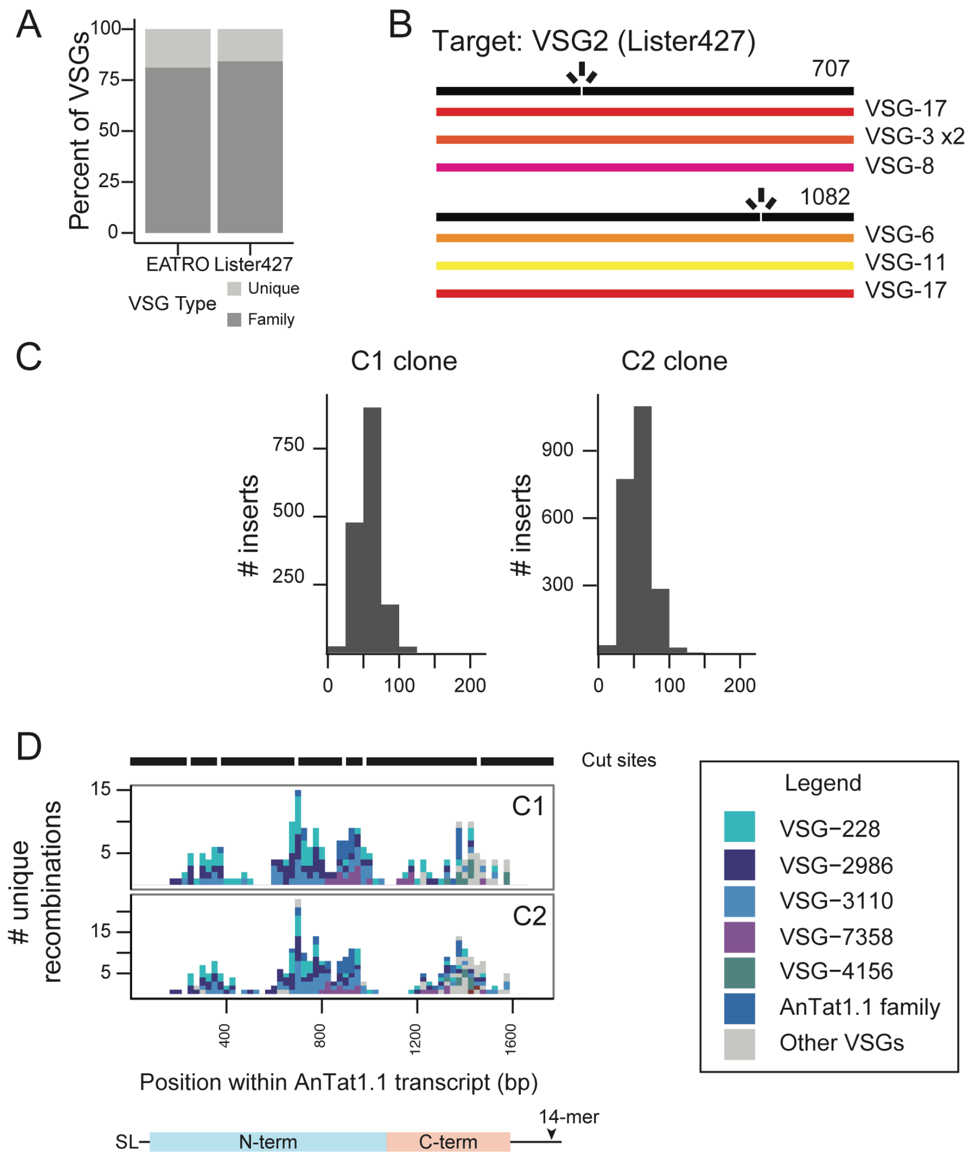

Supplemental Figure 3. DNA breaks result in switchers if a homologous donor is not present
A) Quantification of the types of VSGs within the VSGnomes from EATRO1125 and Lister427 Parasites using the UCLUST greedy clustering algorithm. The Lister427 VSGnome has 4 VSGs which are perfectly duplicated without other family members. B) A schematic showing identified switchers following a Cas9-induced double strand break within VSG-2 at locations 707 and 1082. C) Histograms of donor VSG insertion lengths identified in all mosaic VSG reads from Figure 2A). The insert length only includes newly inserted sequence and does not include recombination sites. Clone C1 and C2 sequenced via VSG-AMP-seq are shown separately. The limit of detection for an insertion is approximately 200 bp. D) A histogram with a summary of the unique recombination sites found within guide-induced break for each clone. Cut sites are indicated above the histograms as gaps within the black line. The midpoint of the perfect homology between AnTat1.1 and the donor VSG at the recombination site is plotted. If a mosaic sequence matched >1 potential donor VSG, the average recombination position was plotted. The legend for the donor VSG colors is to the right. SL = 5′ splice leader sequence, 14-mer = 3′ sequence conserved in all VSG transcripts

bioRxiv preprint doi: https://doi.org/10.1101/2024.03.22.582209; this version posted August 30, 2024. The copyright holder for this preprint (which was not certified by peer review) is the author/funder, who has granted bioRxiv a license to display the preprint in perpetuity. It is made available under aCC-BY 4.0 International license.

1199

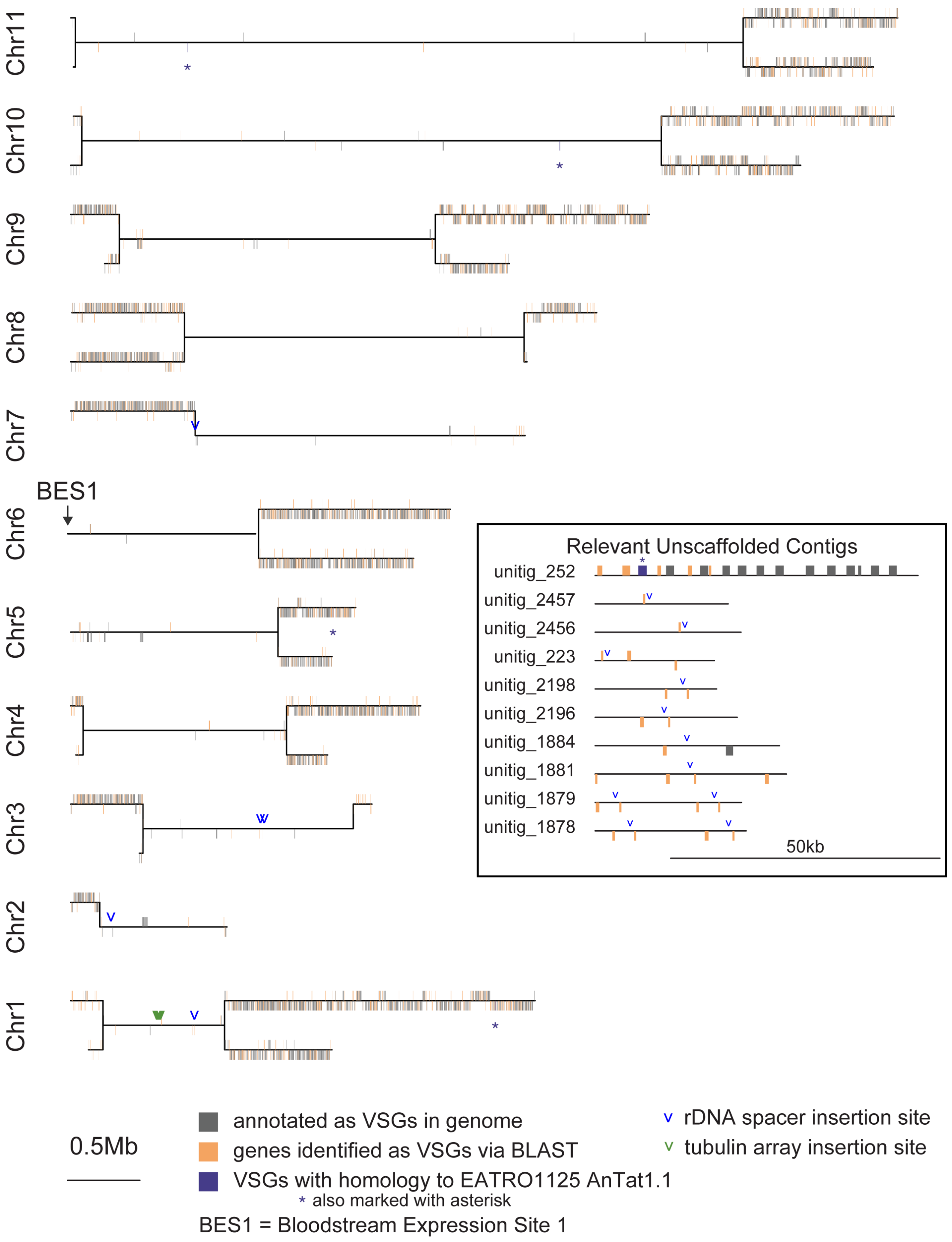

Supplemental Figure 4. The Lister427 VSG annotated genome

The megabase chromosomes from Müller et al.[23] VSGs annotated in the genome are plotted in gray. Unknown genes identified as VSGs via BLAST are plotted in yellow. Details of how these VSGs were identified are detailed in Methods. AnTat1.1 homologous family members are colored purple and marked with an asterisk. Insertion sites for the VSG-228 are denoted by arrows at the insertion location. Inset are the unitigs which are unscaffolded and harbor a copy of the AnTat1.1 family member or a potential insertion site. Bloodstream Expression Site 1 (BES1) is on the 5' end of chromosome 6 and is marked by an arrow.

bioRxiv preprint doi: https://doi.org/10.1101/2024.03.22.582209; this version posted August 30, 2024. The copyright holder for this preprint (which was not certified by peer review) is the author/funder, who has granted bioRxiv a license to display the preprint in perpetuity. It is made available under aCC-BY 4.0 International license.

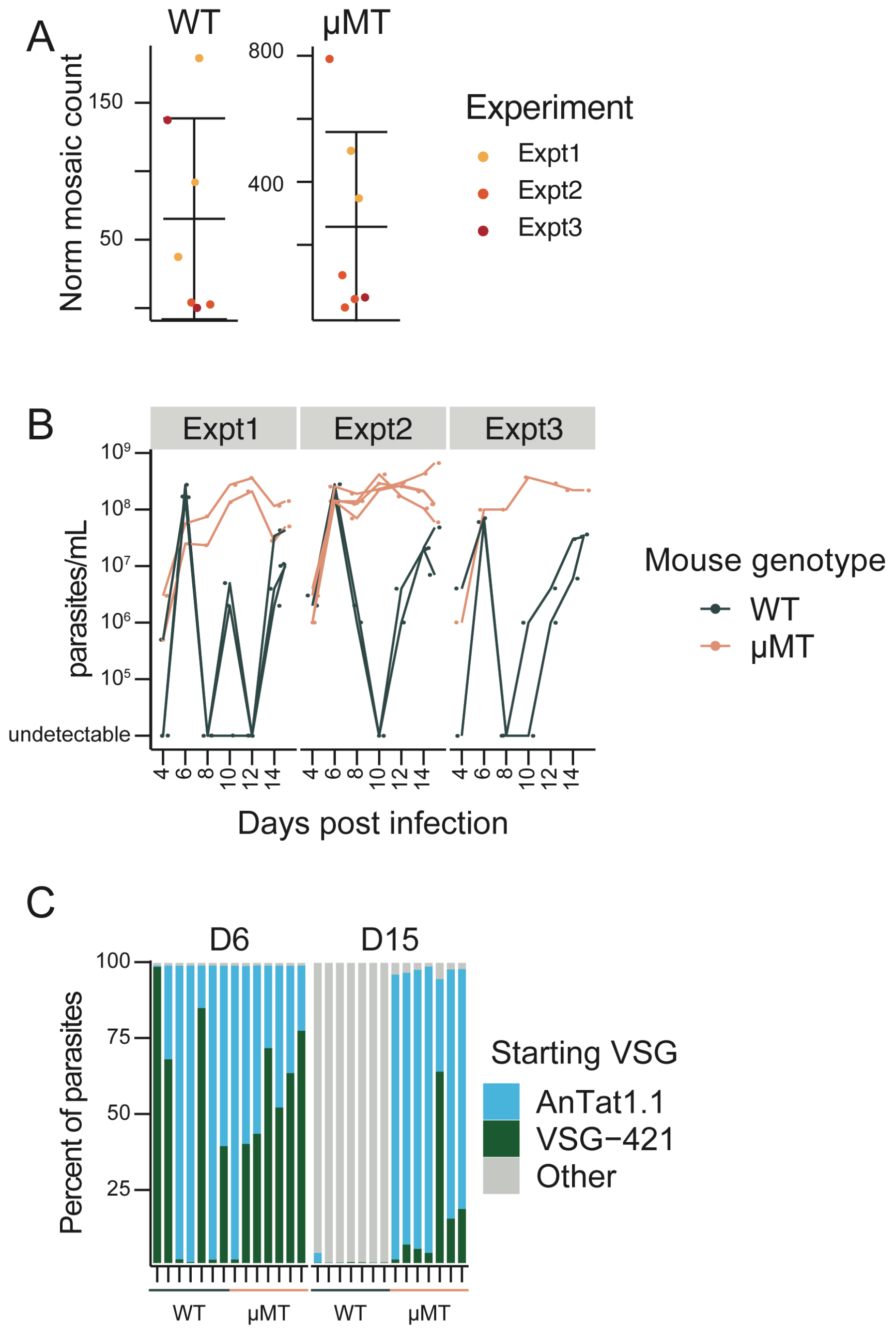

Supplemental Figure 5. Mouse infection extended data

A) Quantification of the number of recombination events detected per mouse. Each mouse was normalized to the number of total consolidated, aligned, and unanchored reads compared to the consolidated, aligned, unanchored read count for one mouse to control for sequencing depth. This was performed separately for each genotype. Mean ± s.d. B) A time course of the parasitemia for the mouse infections. Blood was harvested every two days. C) Quantification of the percent of parasites expressing the starting VSG at D6 and D15 post-infection as quantified by VSG-seq.

bioRxiv preprint doi: https://doi.org/10.1101/2024.03.22.582209; this version posted August 30, 2024. The copyright holder for this preprint (which was not certified by peer review) is the author/funder, who has granted bioRxiv a license to display the preprint in perpetuity. It is made available under aCC-BY 4.0 International license.

Table 1 Guide and VSG-AMP-seq primers

Guide Primers		
Primer Name	Sequence	
AnTat1.1_243	GAAATTAATACGACTCACTATAGGATTCAAAAACGGCCAAACGCCGTTTTAGAGCTAGAAATAGC	
AnTat1.1_369R	GAAATTAATACGACTCACTATAGGGGCGTAAATTAACAGTGTCGGTTTTAGAGCTAGAAATAGC	
AnTat1.1_694	GAAATTAATACGACTCACTATAGGAGTACAGACCCAGAAGCCAGGTTTTAGAGCTAGAAATAGC	
AnTat1.1_894	GAAATTAATACGACTCACTATAGGACGCCGGTGTCGCAGCTAAACGTTTTAGAGCTAGAAATAGC	
AnTat1.1_978R	GAAATTAATACGACTCACTATAGGGTCGTTGGCTGCTTGGAGTTGTTTTAGAGCTAGAAATAGC	
AnTat1.1_1459	GAAATTAATACGACTCACTATAGGACCAATCCAGAAAAGTGCAAGTTTTAGAGCTAGAAATAGC	
G00	AAAAGCACCGACTCGGTGCCACTTTTTCAAGTTGATAACGGACTAGCCTTATTTTAACTTGCTATTTCTAGCTCTAAAAC	
Annealed Guides for T7-sgRNA insertion		
Fragment Name	FWD	REV
AnTat1.1_243	AGGGATTCAAAAACGGCCAAACGCC	AAACGGCGTTTGGCCGTTTTTGAAT
AnTat1.1_369R	AGGGGGCGTAAATTAACAGTGTCG	AAACCGACACTGTTAATTTACGCC
AnTat1.1_694	AGGGAGTACAGACCCAGAAGCCAG	AAACCTGGCTTCTGGGTCTGTACT
AnTat1.1_894	AGGGACGCCGGTGTCGCAGCTAAAC	AAACGTTTAGCTGCGACACCGGCGT
AnTat1.1_978R	AGGGGTCGTTGGCTGCTTGGAGTT	AAACAACTCCAAGCAGCCAACGAC
AnTat1.1_1459	AGGGACCAATCCAGAAAAGTGCAA	AAACTTGCACTTTTCTGGATTGGT
VSG-2_707	AGGGACCAACGGCCTCGGCAAAAG	AAACCTTTTGCCGAGGCCGTTGGT
VSG-2_1082	AGGGCCAGTGGCGCAAAACCTGGT	AAACACCAGGTTTTGCGCCACTGG
DNase Verification Primers		
Primer Name	FWD	REV
HSP-70 (Tb927.11.11330)	AGAACACTATCAATGACCCCAAC	CCATGCCCTGGTACATCT
Hyg	ACAGCGGTCATTGACTGGAG	ATTTGTGTACGCCCGACAGT
VSG-AMP-seq Primers		
Primer Name	Sequence	
All-VSG-3'UTR	GTGTTAAAATATATC	

bioRxiv preprint doi: https://doi.org/10.1101/2024.03.22.582209; this version posted August 30, 2024. The copyright holder for this preprint (which was not certified by peer review) is the author/funder, who has granted bioRxiv a license to display the preprint in perpetuity. It is made available under aCC-BY 4.0 International license.

Y-adapter%	[Phos]GATCGGAAGAGC*C*A
A01^	AATGATACGGCGACCACCGAGATCTACACTAGATCGC(N:25252525)(N)(N)(N)(N)(N)(N)(N)(N)(N)(N)(N)(N)(N)(N)(N)(N)(N)(N)(N)(N)(N)(N)(N)(N)ACACTCTTTCCCTACACGACGCTCTTCCGATC*T
A03^	AATGATACGGCGACCACCGAGATCTACACTATCCTCT(N:25252525)(N)(N)(N)(N)(N)(N)(N)(N)(N)(N)(N)(N)(N)(N)(N)(N)(N)(N)(N)(N)(N)(N)(N)(N)ACACTCTTTCCCTACACGACGCTCTTCCGATC*T
A04^	AATGATACGGCGACCACCGAGATCTACACAGAGTAGA(N:25252525)(N)(N)(N)(N)(N)(N)(N)(N)(N)(N)(N)(N)(N)(N)(N)(N)(N)(N)(N)(N)(N)(N)(N)(N)ACACTCTTTCCCTACACGACGCTCTTCCGATC*T
A05^	AATGATACGGCGACCACCGAGATCTACACGTAAGGAG(N:25252525)(N)(N)(N)(N)(N)(N)(N)(N)(N)(N)(N)(N)(N)(N)(N)(N)(N)(N)(N)(N)(N)(N)(N)(N)ACACTCTTTCCCTACACGACGCTCTTCCGATC*T
A06^	AATGATACGGCGACCACCGAGATCTACACACTGCATA(N:25252525)(N)(N)(N)(N)(N)(N)(N)(N)(N)(N)(N)(N)(N)(N)(N)(N)(N)(N)(N)(N)(N)(N)(N)(N)ACACTCTTTCCCTACACGACGCTCTTCCGATC*T
A07^	AATGATACGGCGACCACCGAGATCTACACAAGGAGTA(N:25252525)(N)(N)(N)(N)(N)(N)(N)(N)(N)(N)(N)(N)(N)(N)(N)(N)(N)(N)(N)(N)(N)(N)(N)(N)ACACTCTTTCCCTACACGACGCTCTTCCGATC*T
A08^	AATGATACGGCGACCACCGAGATCTACACCTAAGCCT(N:25252525)(N)(N)(N)(N)(N)(N)(N)(N)(N)(N)(N)(N)(N)(N)(N)(N)(N)(N)(N)(N)(N)(N)(N)(N)ACACTCTTTCCCTACACGACGCTCTTCCGATC*T
A09^	AATGATACGGCGACCACCGAGATCTACACGACATTGT(N:25252525)(N)(N)(N)(N)(N)(N)(N)(N)(N)(N)(N)(N)(N)(N)(N)(N)(N)(N)(N)(N)(N)(N)(N)(N)ACACTCTTTCCCTACACGACGCTCTTCCGATC*T
A10^	AATGATACGGCGACCACCGAGATCTACACACTGATGG(N:25252525)(N)(N)(N)(N)(N)(N)(N)(N)(N)(N)(N)(N)(N)(N)(N)(N)(N)(N)(N)(N)(N)(N)(N)(N)ACACTCTTTCCCTACACGACGCTCTTCCGATC*T
P5_2	AATGATACGGCGACCACCGAGATCTACAC
P701	CAAGCAGAAGACGGCATACGAGATTCGCCTTAGTGACTGGAGTCCTCTCTATGGGCAGTCGGTGA
P703	CAAGCAGAAGACGGCATACGAGATTTCTGCCTGTGACTGGAGTCCTCTCTATGGGCAGTCGGTGA
P704	CAAGCAGAAGACGGCATACGAGATGCTCAGGAGTGACTGGAGTCCTCTCTATGGGCAGTCGGTGA
P705	CAAGCAGAAGACGGCATACGAGATAGGAGTCCGTGACTGGAGTCCTCTCTATGGGCAGTCGGTGA

bioRxiv preprint doi: https://doi.org/10.1101/2024.03.22.582209; this version posted August 30, 2024. The copyright holder for this preprint (which was not certified by peer review) is the author/funder, who has granted bioRxiv a license to display the preprint in perpetuity. It is made available under aCC-BY 4.0 International license.

P706	CAAGCAGAAGACGGCATACGAGATCATGCCTAGTGACTGGAGTCCTCTCTATGGGCAGTCGGTGA
P707	CAAGCAGAAGACGGCATACGAGATGTAGAGAGGTGACTGGAGTCCTCTCTATGGGCAGTCGGTGA
P708	CAAGCAGAAGACGGCATACGAGATCCTCTCTGGTGACTGGAGTCCTCTCTATGGGCAGTCGGTGA
P709	CAAGCAGAAGACGGCATACGAGATATCACGGTGTGACTGGAGTCCTCTCTATGGGCAGTCGGTGA
P710	CAAGCAGAAGACGGCATACGAGATCGATGTGCGTGACTGGAGTCCTCTCTATGGGCAGTCGGTGA
AnTat1.1_1F[&]	CCTCTCTATGGGCAGTCGGTGAT$(N)_{0-7}$CGCAAACACTACAACGAGCC
AnTat1.1_2F[&]	CCTCTCTATGGGCAGTCGGTGAT$(N)_{0-7}$CAGAATGCGACACGGAAAGC
AnTat1.1_3F[&]	CCTCTCTATGGGCAGTCGGTGAT$(N)_{0-7}$ACGCAGGCGGCTTCAAAACA
AnTat1.1_4F[&]	CCTCTCTATGGGCAGTCGGTGAT$(N)_{0-7}$AACAGCCGCAGCAACCAAAC
AnTat1.1_0R[&]	CCTCTCTATGGGCAGTCGGTGAT$(N)_{0-7}$GGCCACAAATGCGGCAGAAAC
AnTat1.1_1R[&]	CCTCTCTATGGGCAGTCGGTGAT$(N)_{0-7}$GCCATAAGCTGCGGTTTCGT
AnTat1.1_2R[&]	CCTCTCTATGGGCAGTCGGTGAT$(N)_{0-7}$GTTGTGTATGGTTAGCAGGC
AnTat1.1_3R[&]	CCTCTCTATGGGCAGTCGGTGAT$(N)_{0-7}$CTTGTATTTTGTGCGTGGCG
Index1[%]	ATCACCGACTGCCCATAGAGAGGACTCCAGTCAC
Read2[%]	GTGACTGGAGTCCTCTCTATGGGCAGTCGGTGAT
Miscellaneous Additional Primers	
Name	Sequence
SL-FWD	ACAGTTTCTGTACTATATTG
SP6-14mer-REV	GATTTAGGTGACACTATAGTGTTAAAATATATC
AnTat1.1 Sanger Sequencing Primer	AGAGAATACTAAGCTAGTTGGC
Pan AnTat1.1family FWD	ACTACACCCACAACAAGCTCTA

[a] * = indicates a phosphorothioate bond modification
[b] [Phos] = 5’ phosphorylation
[c] ^ = hand mixing
[d] & = machine mixing
[e] % = HPLC purification
